# Selectivity of odorant-binding proteins from the southern house mosquito tested against physiologically relevant ligands

**DOI:** 10.3389/fphys.2015.00056

**Published:** 2015-02-27

**Authors:** Jiao Yin, Young-Moo Choo, Hongxia Duan, Walter S. Leal

**Affiliations:** Department of Molecular and Cellular Biology, University of California, DavisDavis, CA, USA

**Keywords:** CquiOBP1, CquiOBP2, CquiOBP5, *Culex quinquefasciatus*, docking

## Abstract

As opposed to humans, insects rely heavily on an acute olfactory system for survival and reproduction. Two major types of olfactory proteins, namely, odorant-binding proteins (OBPs) and odorant receptors (ORs), may contribute to the selectivity and sensitivity of the insects' olfactory system. Here, we aimed at addressing the question whether OBPs highly enriched in the antennae of the southern house mosquito, *Culex quinquefasciatus*, contribute at least in part to the selective reception of physiologically relevant compounds. Using a fluorescence reporter and a panel of 34 compounds, including oviposition attractants, human-derived attractants, and repellents, we measured binding affinities of CquiOBP1, CquiOBP2, and CquiOBP5. Based on dissociation constants, we surmised that CquiOBP2 is a carrier for the oviposition attractant skatole, whereas CquiOBP1 and CquiOBP5 might transport the oviposition pheromone MOP, a human-derived attractant nonanal, and the insect repellent picardin. Binding of these three ligands to CquiOBP1 was further analyzed by examining the influence of pH on apparent affinity as well as by docking these three ligands into CquiOBP1. Our findings suggest that CquiOBP1 might discriminate MOP from nonanal/picaridin on the basis of the midpoint transition of a pH-dependence conformational change, and that MOP is better accommodated in the binding cavity than the other two ligands. These findings, along with previous experimental evidence suggesting that CquiOBP1 does not detect nonanal *in vivo*, suggest that OBP selectivity may not be clearly manifested in their dissociation constants.

## Introduction

In insects, olfaction is essential for survival and reproduction (Leal, 2012); smell is undoubtedly their most important sensory modality. Humans, by contrast, rely more on vision (sight) than smell (Sell, 2014). Consequently, there are major differences but also commonalities between human and insect olfaction. For the reception of olfactory signals, be it an insect pheromone or the smell of food spoiled for human consumption, the odorant molecules must be transported from the external environment to receptors embedded in the membrane of olfactory receptor neurons (ORNs; also called olfactory sensory neurons, OSN). Humans use only one odorant-binding protein (OBP) to carry odorants to 350–400 types of olfactory receptors (ORs, also named odorant receptors) (Sell, 2014). By contrast, insect genomes carry as many *OBP* as *OR* genes (Sell, 2014). It is now accepted that insect OBPs solubilize odorants (ligands), help transport hydrophobic molecules through the aqueous environment of a lymph surrounding the neurons, and contribute to the sensitivity of the olfactory system (Leal, 2013). However, if these were the only roles for OBPs, why do the genome of some species have as many as 50 *OBP* genes? (Leal, 2013) Theoretically, these roles could be performed with a single OBP as in humans (Sell, 2014).

Diseases transmitted by mosquitoes destroy more lives on a year basis than war, terrorism, gun violence, and other human maladies combined (Leal, 2014). Understanding mosquito olfaction may lead to alternative means of controlling mosquito populations as well as to user-friendly chemicals to reduce mosquito bites and, consequently, transmission of diseases. With the advent of RNA-Seq, it is now possible to examine the expression patterns of the entire repertoire of olfactory genes. For example, differential expression analysis of olfactory genes in the southern house mosquito *Culex quinquefasciatus—*a vector of the West Nile virus in the United States - showed that 24 *OBP* have higher transcript levels in antennae than in non-olfactory tissues, whereas 21 *OBP* transcripts were enriched in legs compared to antennae thus questioning their significance for olfaction (Leal et al., 2013). As pointed out earlier, the *OBP* gene family includes in addition to olfactory proteins, non-olfactory proteins, which might be carriers of other non-olfactory hydrophobic ligands. Although only a fraction of the *OBP* in an insect's genomic pool might be involved in olfaction, the ratio of putative *OBP* to *OR* genes suggests that OBP may play other crucial roles for olfaction. In *Cx. quinquefasctiatus*, for example, there are 176 putative ORs and 24 putative OBPs (Leal et al., 2013). As there are multiple pseudogenes or non-functional ORs, the actual OR/OBP ratio might be lower than 7.3:1. Thus, if OBPs differ in their ability to carry and deliver ligands to receptors they might contribute to the selectivity of the insect's olfactory system.

Selectivity of OBPs is typically investigated by comparing their binding affinities for physiologically relevant compounds, i.e., by inferring their ability to carry preferentially certain ligands than others. For example, binding affinity of an OBP from the malaria mosquito *Anopheles funestus*, AfunOBP1, was tested against a panel of compounds that generated electroantennographic (EAG) responses, with 2-undecanone being identified as the best ligand (Xu et al., 2010). Likewise, the orthologous protein from *An. gambiae*, AgamOBP1, was demonstrated to selectively bind indole (Biessmann et al., 2010). Albeit biased due to the small set of ligands tested, these studies suggest that OBP are selective. Even when multiple ligands bind to the same OBP with apparently high affinity, there seem to be other factors contributing to selectivity.

Our biochemical, biophysical and structural studies suggest that a pH-mediated conformational change leads to the delivery of odorant to receptors, although there is another school that support receptor activation by OBP-odorant complexes (Leal, 2013). Recently, we have demonstrated with the pheromone-binding protein from the silkworm moth, BmorPBP1, that the midpoint in the pH transition is influenced by the ligand (Damberger et al., 2013). In short, compounds that bind to an OBP and leads to a very low midpoint transition are trapped by OBPs, whereas those generating a high midpoint transition may be dropped away from the receptor. Therefore, only those ligands with an appropriate midpoint transition are protected from degradation and transported to their cognate receptors.

This research was designed to investigate the potential role of OBPs on the selectivity of the southern house mosquito olfactory system by a three-prong approach. First, we selected 5 OBPs with the highest differential transcript levels in antennae (vs. legs) to clone, express, and test their binding affinity against a panel of 34 physiologically relevant compounds. Then, we studied the effect of pH on the binding of the mosquito oviposition pheromone to CquiOBP1. Lastly, we docked into CquiOBP1 the ligands with the highest affinities to study their interactions in the binding site.

## Materials and methods

### RNA extraction and cloning of CquiOBPs

Mosquitoes were raised, as previously described (Xu et al., 2014). One thousand antennae from 4 to 6-day-old female mosquitoes after blood-fed were dissected and collected in DEPC/EtOH (1:1) on ice using a stereo microscope (Zeiss, Stemi DR 1663, Germany). Total RNA was extracted using TRIzol reagent (Invitrogen, Carlsbad, CA). cDNA was synthesized from 1 μg of total RNA using RT-for-PCR kit according to the manufacturer's instructions (Clontech, Mountain View, CA). To obtain full-length coding sequences, PCR was performed using the following gene specific primers (underline is restriction enzyme site):

CquiOBP2-Fw (*Kpn*I): 5′- GGGGTACCCGAGGAACCGAGGCGAGATGCT -3′;

CquiOBP2- Rv (*Bam*HI): 5′- CGGGATCCCGTCAGGGCAAAAAGTAGTGCAC -3′;

CquiOBP3-Fw (*Kpn*I): 5′- GGGGTACCCGGAGACTTACCGCCACCGAGA -3′;

CquiOBP3- Rv (*Bam*HI): 5′- CGGGATCCCG CTATAGGCAATTTGGAAAGAG -3′;

CquiOBP5-Fw (*Kpn*I): 5′- GGGGTACCCATGACGGTGGCCACCTGGTTA-3′;

CquiOBP5-Rv (*Bam*HI): 5′- CGGGATCCCGTCAAAACAGGTAATAGTGGAC -3′;

CquiOBP11-Fw (*Kpn*I): 5′- GGGGTACCCAAAGCCACCGTCGAGCAGATG -3′;

CquiOBP11-Rv (*Bam*HI): 5′- CGGGATCCCGCTAGGGAAACACAAACTTGGG -3′;

PCR reactions were carried out using Advantage GC 2 PCR Kit (Clontech, Mountain View, CA). PCR products were purified by QIAquick gel extraction kit (Qiagen, Valencia, CA) and then cloned into pGEM-T vector (Promega, Madison, WI). After screening colonies, plasmids were extracted using the QIAprep Spin Miniprep kit (Qiagen) and sequenced by ABI 3730 automated DNA sequencer at Davis Sequencing (Davis, CA).

One microgram of pET-22b(+) vector (EMD Chemicals,Gibbstown, NJ) was digested with 6 U of *Msc*I (New England Biolabs, Ipswich, MA) at 37°C for 3 h. After purification of DNA by QIAquick PCR Purification Kit (Qiagen, Valencia, CA) the vector was digested with 7 U of *Bam*HI (New England Biolabs) at 37°C for 3 h and subsequently gel-purified by QIAquck Gel Extraction Kit (Qiagen). Initially 10 μg of screened plasmids (CquiOBPs) were digested with 40 U of *Kpn*I (New England Biolabs) at 37°C for 3 h, purified by QIAquick PCR Purification Kit, blunted by T4 DNA polymerase (New England Biolabs) with dNTP, and purified again by QIAquick PCR Purification Kit. Then, the DNA was digested with 20 U of *Bam*HI at 37°C for 3 h and, gel- purified by QIAquick Gel Extraction Kit, and ligated into prepared pET-22b(+) vector by T4 DNA ligase (Promega, Madison, WI).

CquiOBP1 was expressed following our previous protocol (Leal et al., 2008). Proteins in the periplasmic fraction were extracted with 10 mM Tris-HCl, pH 8 by three cycles of freeze-and-thaw (Leal, 2000) and centrifuging at 16,000 × g to remove debris. The supernatant was loaded on a Hiprep TM DEAE 16/10 column (GE Healthcare Life Sciences, Piscataway, NJ). All separations by ion-exchange chromatography were done with a linear gradient of 0–500 mM NaCl in 10 mM Tris-HCl, pH 8. Fractions containing a target protein were further purified on a 20-ml Q-Sepharose Hiprep TM16/10 column (GE Healthcare) and, subsequently, on a Mono-QH 10/10 column (GE Healthcare). OBP fractions were concentrated by using Centriprep-10 (Millipore, Billerica, MA) and loaded on a Superdex-75 26/60 gel-filtration column (GE Healthcare) pre-equilibrated with 150 mM NaCl and 20 mM Tris-HCl, pH 8. Highly purified protein fractions were concentrated by Centricon-10, desalted on four 5-ml HiTrap desalting columns (GE Healthcare) in tandem with water and stored at −80°C until use. CquiOBP2, CquiOBP3, and CquiOBP5 were expressed following the same protocol for CquiOBP1 and induced with 1 mM IPTG after optimization attempts with 0.1, 0.4, 0.7, 1, 1.5, and 2 mM IPTG. CquiOBP2, CquiOBP3, and CquiOBP5 were expressed at 28°C, 32°C, and 37°C. Purification protocol was identical to those described for CquiOBP1.

### Fluorescence binding assays

Binding affinity (Ban et al., 2002) was tested with a panel of 34 compounds, including attractants, a mosquito oviposition pheromone, repellents, terpenoids and other putative mosquito attractants or repellents. Unless otherwise specified, they were purchased from Sigma-Aldrich/Fluka/Acros (St. Louis, MO). Repellents: DEET (*N,N*-diethyl-3-methylbenzamide), PMD (p-mentan-3,8-diol) (Bedoukian Research, Inc.), picaridin (butan-2-yl 2-(2-hydroxyethyl)piperidine-1-carboxylate), IR3535 (ethyl 3-[acetyl(butyl)amino]propanoate) (gifts from Dr. Kamal Chauhan, USDA, ARS, Beltsville), (±)-citronellal, and ethyl 2-phenylacetate. Oviposition attractants: (5*R*,6*S*)-6-acetoxy-5-hexadecanolide, MOP (Bedoukian Research, Inc.), skatole, indole, 4-methylphenol, and 4-ethylphenol. Human derived compounds: nonanal, 6-methyl-5-hepten-2-one (sulcatone). Terpenoids and other putative attractants/repellents: linalool, linalool oxide, eucalyptol, camphor, fenchone, thujone, geranyl acetate, eugenol, (Z)-3-hexenyl acetate, 1-octen-3-ol, 1-octyn-3-ol, 3-octanol, 1-hexanol, methylcyclohexanol, methyl salicylate, cyclohexanone, acetophenone, benzaldehyde, 3,5-dimethoxybenzene, 1,2-dimethoxybenzene, and 2-phenylethanol.

Fluorescence spectra were recorded in a right angle configuration on a spectrofluorimeter (RF-5301, Shimadzu, Kyoto, Japan) at room temperature using a 1 cm light path fluorimeter quartz cuvette. Slit widths of 10 nm were selected for both excitation and emission. N-phenyl-1-naphthylamine (NPN, or 1-NPN) (Ban et al., 2002) was dissolved in methanol to yield a 1 mM stock solution. The binding affinity for NPN was determined by adding aliquots of NPN into a 10 μg/ml (microgram) protein sample. NPN was excited at 337 nm and emission spectra were recorded at high speed scanning between 350 and 500 nm. All ligands used in competition experiments were dissolved in spectrophotometric grade methanol. Binding data were collected in three independent measurements. Bound ligand was evaluated from fluorescence intensity assuming that the protein was 100% active, with a stoichiometry of 1:1 (protein: ligand) at saturation. The K_NPN_ values were estimated using Prism 5 (GraphPad Software, Inc., La Jolla, CA) by non-linear regression for a unique site of binding. The curves were linearized using Scatchard plots. Dissociation constants of the competitors were calculated according to Campanacci et al. (2001) from the corresponding IC_50_ values in the equation: K_diss_ = [IC50]/1+[NPN]/K_NPN_, where [NPN] is the free concentration of NPN and K_NPN_ is the dissociation constant of the complex protein/NPN.

### pH-dependent binding assay

To investigate the effects of pH on CquiOBP1–ligand complexes, fluorescence binding assay was measured between CquiOBP1 and three best ligands at different pH values. Buffer were prepared into two different ways: starting from 1M phosphate buffer, pH 4, pH was increased and adjusted by adding sodium hydroxide. Then protein was added to make a solution at each tested pH from 4.5 to 8. Experiments were repeated starting from pH 8 and lowering the pH by adding hydrochloric acid. Each tested solution had 200 mM buffer and 10 μg/ml (microgram) of CquiOBP1. As there were no significant differences in fluorescence data generated at a certain pH regardless of having the pH raised by adding NaOH or lowered by addition of HCl, data were pooled for subsequent analysis.

### Docking assays

Molecular docking was performed with Surflex-dock module in Sybyl vs.7.3 software (Tripos Associates, St. Louis, MO). To determine the suitability of our approach, we first compared the crystal structure of CquiOBP1-MOP complex (Mao et al., 2010) with that obtained by docking MOP back into CquiOBP1. The complex crystal structure of CquiOBP1-MOP was retrieved from the RCSB Protein Data Bank (PDB code: 3OGN). The ligand MOP was extracted from the crystal complex and then was docked into the binding pocket of CquiOBP1 again to determine the suitability of the docking method. Prior to docking we determined the lowest energy conformation for each ligand. Three ligands were optimized by Merck Molecular Force Field MMFF94 force field (Halgren, 1996) and MMFF94 charge to get a lower energy conformation as an initial one for subsequent docking study. Then, Surflex-Dock score (Cscore) was used to determine binding affinities of CquiOBP1 for MOP and the other two best ligands, picaridin and nonanal. Lastly, the scoring function (Cscore) was used to predict the binding affinities of protein/ligand complexes. CScore integrates a number of popular scoring functions for ranking the affinity of ligands bound to the binding site of a protein, and it is more robust and accurate than any single function for evaluating ligand-protein interactions.

## Results and discussion

### Gene cloning and expression

CquiOBP1 is the first olfactory protein identified from mosquitoes by conventional biochemical approach, i.e., isolation, N-terminal sequencing and subsequent cDNA cloning with degenerate primers (Ishida et al., 2002). This approach was biased as leading only to OBPs highly expressed in olfactory tissues. With RNA-Seq we have recently identified the complete repertoire of putative OBPs (Leal et al., 2013). Despite its obvious advantages, RNA-Seq findings need to be confirmed by gene cloning as this bioinformatics approaches may include pseudogenes and other algorithm artifacts. Here, we aimed at cloning *Culex OBP* highly enriched in antennae to subsequently compare their binding to a panel of physiologically relevant compounds. Specifically, we aimed at cloning CquiOBP3, CquiOBP5, CquiOBP2, and CquiOBP11, which are enriched in *Culex* antennae as indicated by the moderated log fold—a ratio of transcript levels in antennae compared to legs (Leal et al., 2013). Their transcript levels were even higher than those for *CquiOBP1*, which has been isolated, cloned (Ishida et al., 2002), ant the protein expressed (Leal et al., 2008) and studied by X-ray crystallography and nuclear magnetic resonance (Mao et al., 2010).

All OBP cDNAs cloned were identical to those reported in GenBank, specifically CquiOBP2: FJ947084, 146 aa residues, including a signal peptide (Petersen et al., 2011) with 22 aa residues; CquiOBP3: FJ947085, 147 aa residues, including a signal peptide of 18 aa residues), CquiOBP5: FJ947087, 143 aa residues; no signal peptide; and CquiOBP11: FJ947091, 144 aa residues, including a 23-aa-long signal peptide. The theoretical isoelectric points (Wilkins et al., 1999) of the mature proteins were: CquiOBP2, 5.33; CquiOBP3, 5.42; CquiOBP5, 5.16; CquiOBP11, 8.52. Considering that CquiOBP11 is not an acidic protein, we selected CquiOBP2, CquiOBP3, and CquiOBP5 for expression, along with CquiOBP1 and subsequent binding studies.

Expression of CquiOBP3 at different temperatures and different IPTG concentrations gave low yields. Even at the best conditions, the yield was so low that the target protein was lost during purification attempts. By contrast, CquiOBP2 and CquiOBP5 were obtained at high yields, as previously reported for CquiOBP1. While large-scale expression of CquiOBP1 and CquiOBP2 gave the highest yields at 28°C, 1–3 h after induction with IPTG, optimal expression of CquiOBP5 was achieved under the same conditions but at 37°C. Highly purified CquiOBP1, CquiOBP2, and CquiOBP5 were used for subsequent binding assays.

### Binding assays

**Figure 1:**
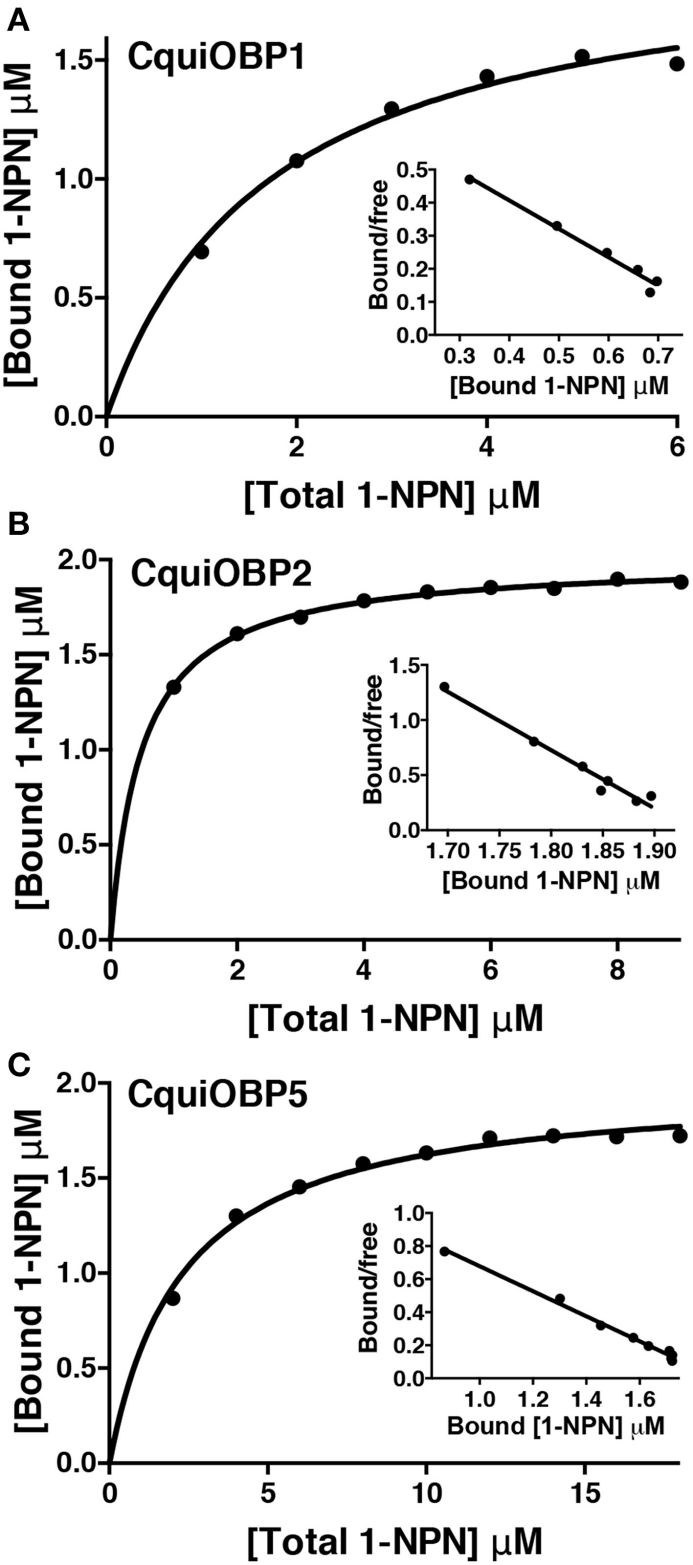
**Titration of OBPs with the fluorescence reporter NPN. (A)** CquiOBP1, **(B)** CquiOBP2, and **(C)** CquiOBP5. The respective Scatcahrd plots are inserted under the curves.

The three OBPs tested show affinity for NPN within the normal range reported in the literature (Ban et al., 2002), i.e., CquiOBP2 (*Kd* = 0.5 ± 0.02 μM), CquiOBP1 (*Kd* = 1.71 ± 0.13 μM), and CquiOBP5 (*Kd* = 2.3 ± 0.19 μM) (Figure 1). Our panel of physiologically relevant compounds was then tested against these OBPs. Compounds that led to significant levels of NPN displacement, as inferred by fluorescence quenching, were further evaluated to determine their dissociation constants. Initially, compounds were screened at final concentrations from 2 to 10 μM (see residual intensity, Table 1). Those leading to significant reduction of fluorescence intensity were evaluated on a dose-dependent manner.

**Table 1:** **Binding affinities of CquiOBPs to a group of compounds**.

**Ligand**	**CquiOBP1**			**CquiOBP2**			**CquiOBP5**		
	**IC_50_**	***Int***	***K_d_***	**IC_50_**	***Int***	***K_d_***	**IC_50_**	***Int***	***K_d_***
DEET	–	82	–	–	96	–	–	92	–
PMD	–	80	–	5.3	35	1.3	13.8	61	9.6
Picaridin	10.2	51	6.4	–	77	–	7.9	45	5.6
IR3535	–	71	–	–	90	–	–	67	–
Skatole	–	75	–	5.6	31	1.4	14.4	62	10.1
Indole	–	86	–	–	87	–	–	80	–
MOP	8.4	48	5.3	–	86	–	10.3	52	7.2
4-Methylphenol	–	73	–	–	85	–	–	90	–
4-Ethylphenol	–	85	–	–	91	–	–	84	–
(*Z*)-3-Hexenyl acetate	–	79	–	–	93	–	–	83	–
1-Octen-3-ol	–	69	–	–	93	–	11.6	59	8.1
3-Octanol	–	87	–	–	78	–	–	85	–
1-Hexanol	–	85	–	–	89	–	–	88	–
(±)-Citronellal	–	65	–	–	88	–	–	90	–
Ethyl 2-phenylacetate	–	92	–	–	87	–	–	91	–
Sulcatone	–	87	–	–	84	–	–	66	–
4-Methylcyclohexanol	–	88	–	–	88	–	–	93	–
Methyl salicylate	–	87	–	–	72	–	–	89	–
Geranyl acetate	–	94	–	–	90	–	–	81	–
Eugenol	–	72	–	–	82	–	10.7	57.5	7.4
Cyclohexanone	–	79	–	–	86	–	–	82	–
Acetophenone	–	88	–	–	92	–	–	87	–
Thujone	–	96	–	–	87	–	–	90	–
Benzaldehyde	–	83	–	–	91	–	–	93	–
3,5-Dimethylphenol	–	84	–	7.4	40	1.9	–	93	–
1,2-Dimethoxybenzene	–	88	–	–	91	–	–	87	–
2-Phenylethanol	–	91	–	–	92	–	–	95	–
Linalool oxide	–	92	–	–	95	–	–	94	–
Linalool	–	91	–	–	97	–	–	86	–
Eucalyptol	–	96	–	–	90	–	–	73	–
Nonanal	10.5	51.4	6.6	–	87	–	8.3	46.5	5.8
Camphor	–	90	–	–	85	–	–	89	–
1-Octyn-3-ol	–	66	–	–	92	–	12.8	62.2	8.9
Fenchone	–	89	–	–	87	–	–	92	–

*Ligands were screened by adding aliquots of each ligand to NPN-OBP solutions so as to make 2–20 μM of ligand (final concentrations). Fluorescence emission (Int) prior to addition of test ligands were normalized to 100. For ligands that caused significant quenching (>20%) with the addition of 10 μM of ligand, titration was continued to calculate their dissociation constants. (–) Denotes low or no binding*.

**Figure 2:**
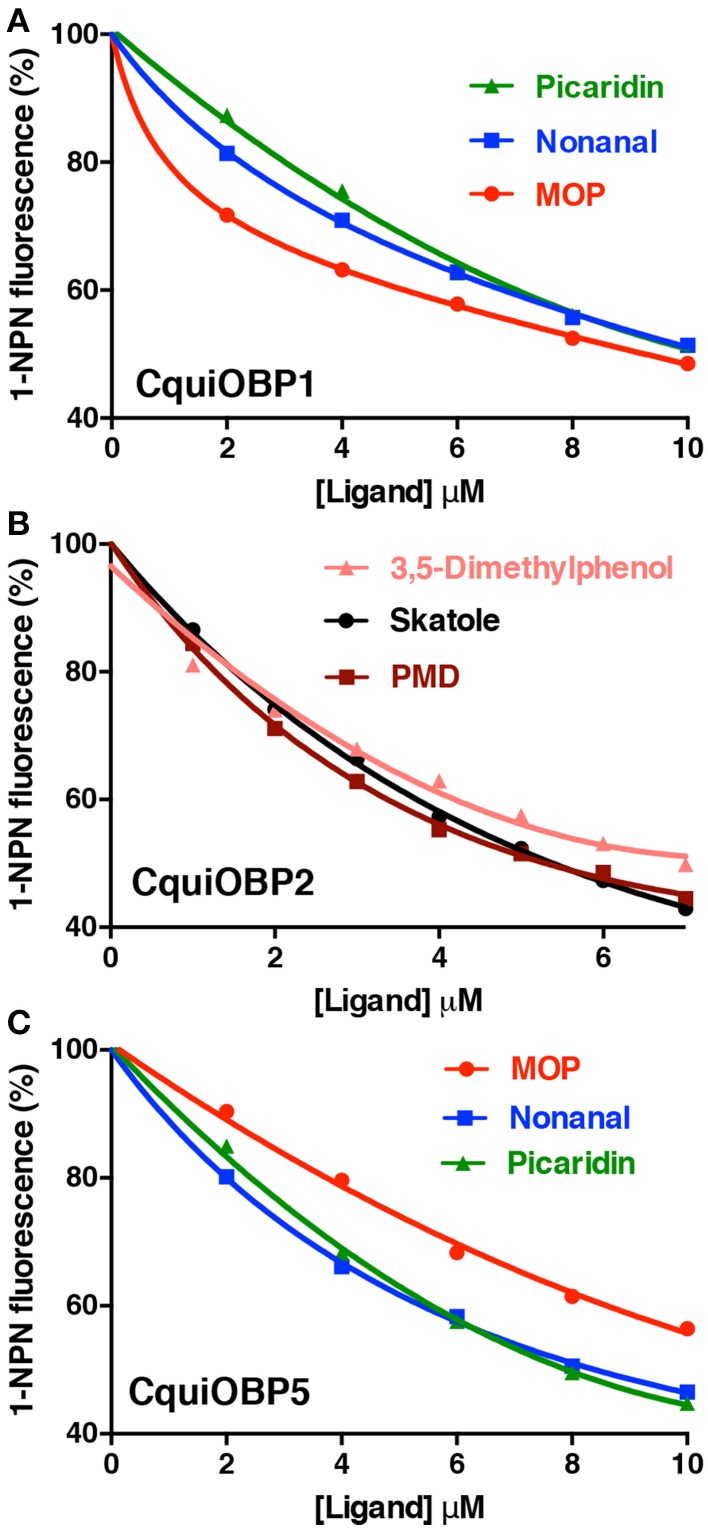
**Titration of NPN displacement by high affinity odorants**. **(A)** Binding of picaridin, nonanal and MOP to CquiOBP1. **(B)** Titration of NPN fluorescence in the presence of CquiOBP2 by 3,5-dimethylphenol, skatole, and PMD. **(C)** Binding of MOP, nonanal, and picaridin to CquiOBP5.

The dissociation constants for the best ligands for CquiOBP1, namely, MOP (Laurence and Pickett, 1982), picaridin, and nonanal, were 5.3, 6.4, and 6.6 μM (Table 1 and Figure 2A). Of notice, the orthologous protein from the yellow fever mosquito, AaegOBP1, bound MOP, but not picaridin (Leal and Leal, 2015). None of the three ligands bound to CquiOBP2, which in turn showed highest affinities for PMD (*Kd* = 1.3 μM), skatole (*Kd* = 1.4 μM), and 3,5-dimethylphenol (*Kd* = 1.9 μM) (Table 1, Figure 2B). Interestingly, three of the best ligands for CquiOBP5 were the same as those for CquiOBP1 (Table 1, Figure 2C). They differ, however, in the order with the human-derived mosquito attractant nonanal and the insect repellent picaridin showing the highest affinity for CquiOBP5, and the oviposition attractant MOP showing the lowest affinity of the three ligands (Table 1). The reverse was observed for CquiOBP1, which showed highest affinity for MOP. These differences in affinity suggest that OBPs might be involved in the selectivity of the mosquito olfactory system. For example, it is very unlikely that CquiOBP1 would be involved in the detection of the oviposition attractant skatole as it does not bind to skatole *in vitro*. By contrast, both CquiOBP2 and CquiOBP5 bind skatole but the large difference in their dissociation constants suggest that CquiOBP2 is more likely to be involved in skatole reception than CquiOBP5. By contrast, it is unlikely that reception of MOP is mediated by CquiOBP2 given MOP cannot displace NPN from this protein (Table 1). Both CquiOBP1 and CquiOBP5 bound MOP, but the higher affinity displayed by CquiOBP1, along with its expression in MOP-detecting sensilla in *Culex* antennae (Leal et al., 2008), suggest that CquiOBP1 is likely to transport MOP *in vivo*. Given our previous findings showing that CquiOBP1 undergo a pH-dependent conformational change (Leal et al., 2008), structural evidence suggesting that this conformational change might be physiologically relevant (Mao et al., 2010), and our recent finding indicating the importance of the pH transition midpoint (Damberger et al., 2013), we next analyzed CquiOBP1 binding affinity at various pH values.

### pH-dependent binding assays

**Figure 3:**
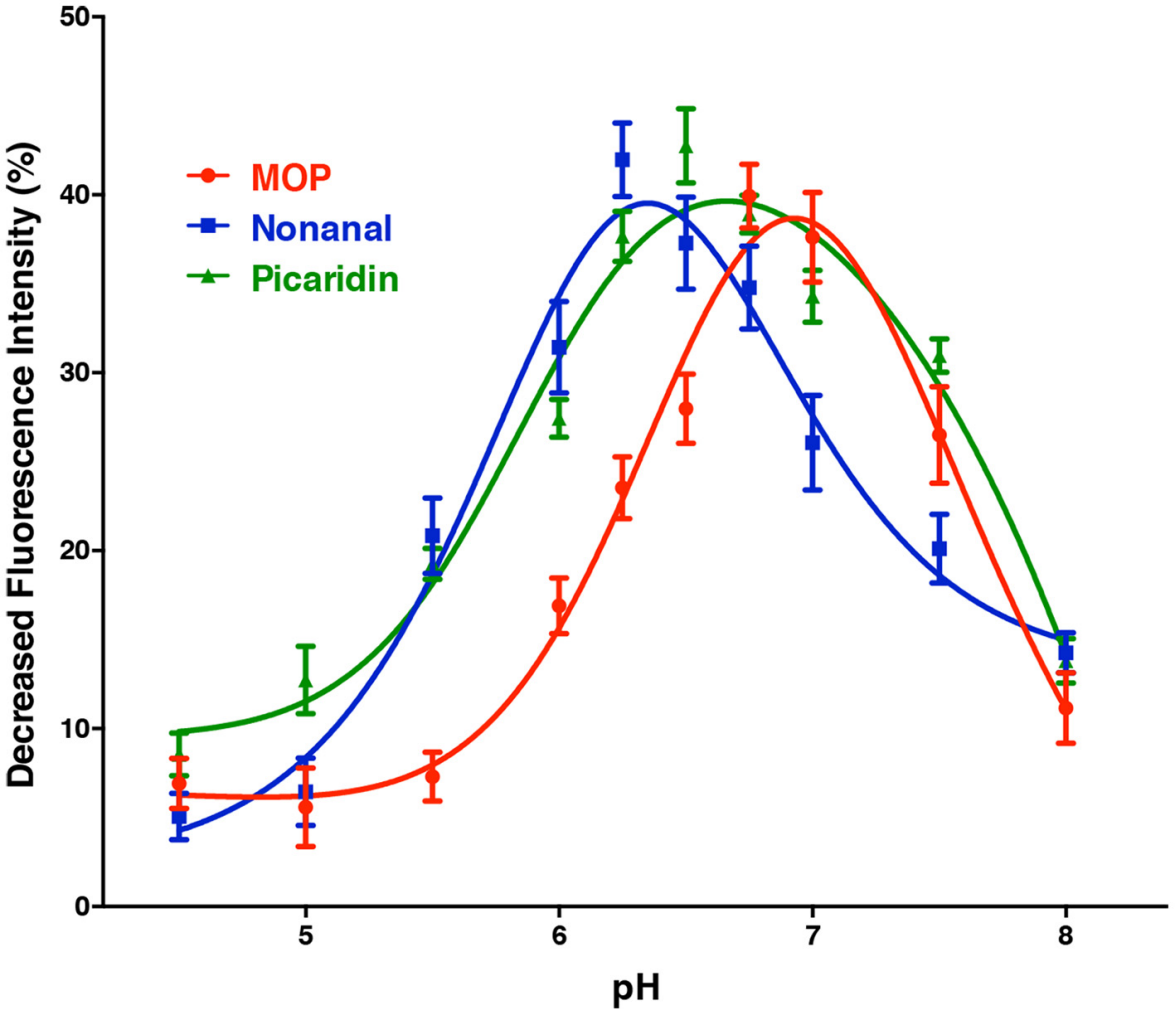
**pH-dependent quenching of fluorescence emission by CquiOBP1-bound NPN**. All curves showed a truncated bell-shape curve, with minimal quenching at low pH and maximal quenching at high pH (6–7). Quenching decreased at pH values higher than 7, i.e., the pH expected for the sensillar lymph (Kaissling, 2009).

The pH dependent curves for CquiOBP1 obtained with the three best ligands showed a nearly bell-shape curve, with three different maxima (Figure 3). The pH of the midpoint transitions was obtained by non-linear fitting, sigmoidal with an equation for four-parameter logistic (4PL) of curve segments between the lowest and highest decrease in fluorescence (the rising part of each curve). The pH values calculated for the midpoint transitions with MOP, picaridin, and nonanal were 6.3, 5.8, and 5.5, respectively. Although the pH at the surface of dendritic membrane is not known, it is predicted that too low midpoint transition may lead to odorants being trapped in OBPs and not being properly delivered. In short, the acidity at the surface of the membrane may not be enough to trigger release of a ligand with too low midpoint transition pH. By considering only the dissociation constants obtained by *in vitro* binding assays (Table 1), one could reasonably argue that CquiOBP1 is involved in reception of nonanal. Previous work with CquiOBP1 knockdown experiments suggested that MOP, but not nonanal, is carried by CquiOBP1 (Pelletier et al., 2010). Mosquitoes with reduced transcripts of CquiOBP1 gave significant lower EAG responses to MOP, but EAG responses to nonanal were not significantly different when comparing wild type and knockdown mosquitoes (Pelletier et al., 2010). Taken together, these findings suggest that, despite displaying a suitable dissociation constant to transport nonanal and picaridin, the midpoint transition pH for CquiOBP1 may be too low to properly deliver these odorants to odorant receptors. Next, we compared how these ligands would interact with CquiOBP1.

### Docking studies

**Figure 4:**
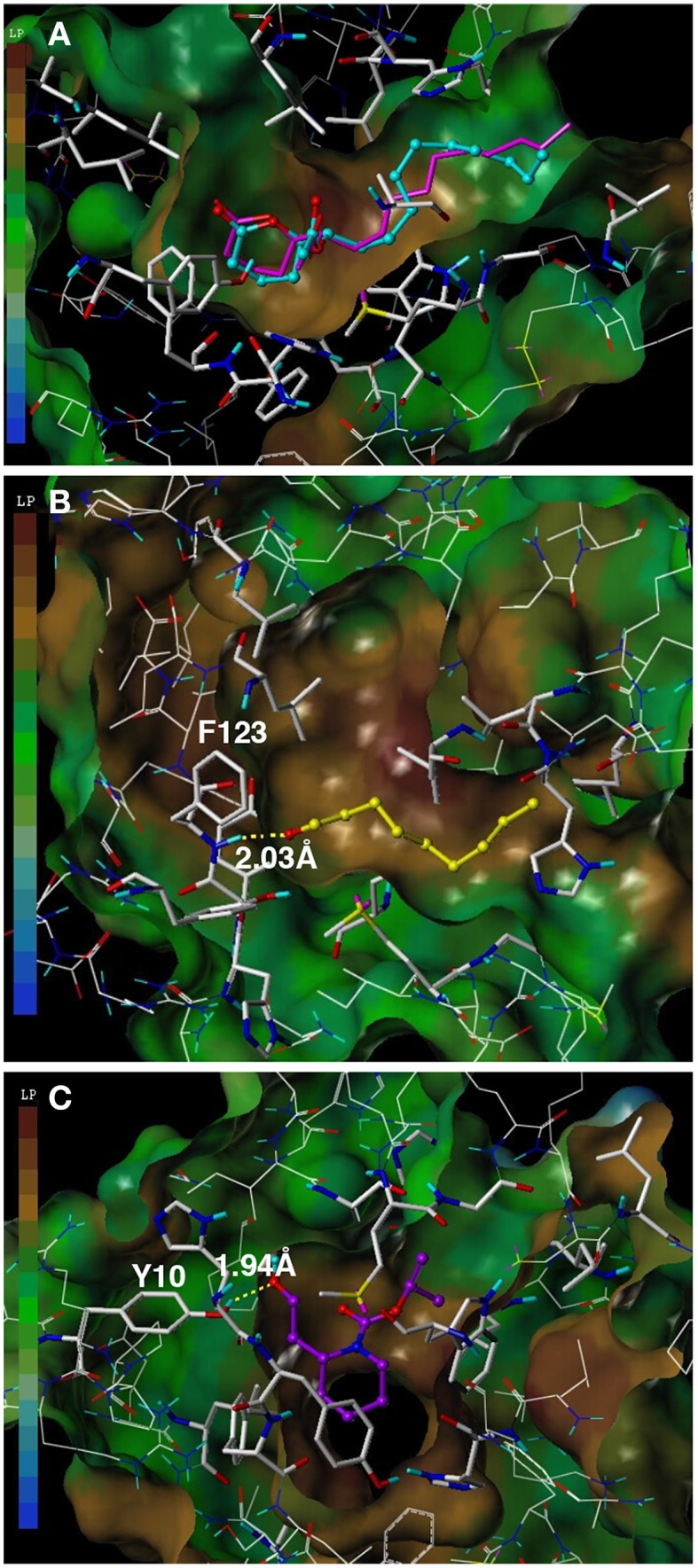
**Docking of ligands into CquiOBP1. (A)** Re-docked MOP (cyan) and MOP as observed in the CquiOBP1-MOP crystal complex (Mao et al., 2010). **(B)** Nonanal bound to the main cavity and stabilized by a hydrogen bond with the main chain of Phe-123. **(C)** Picaridin bound in the same cavity, but having a different orientation and the hydroxyl group forming a hydrogen bond with the side chain of Tyr-10. Figures were generated with Sybyl 7.3.

**Figure 5:**
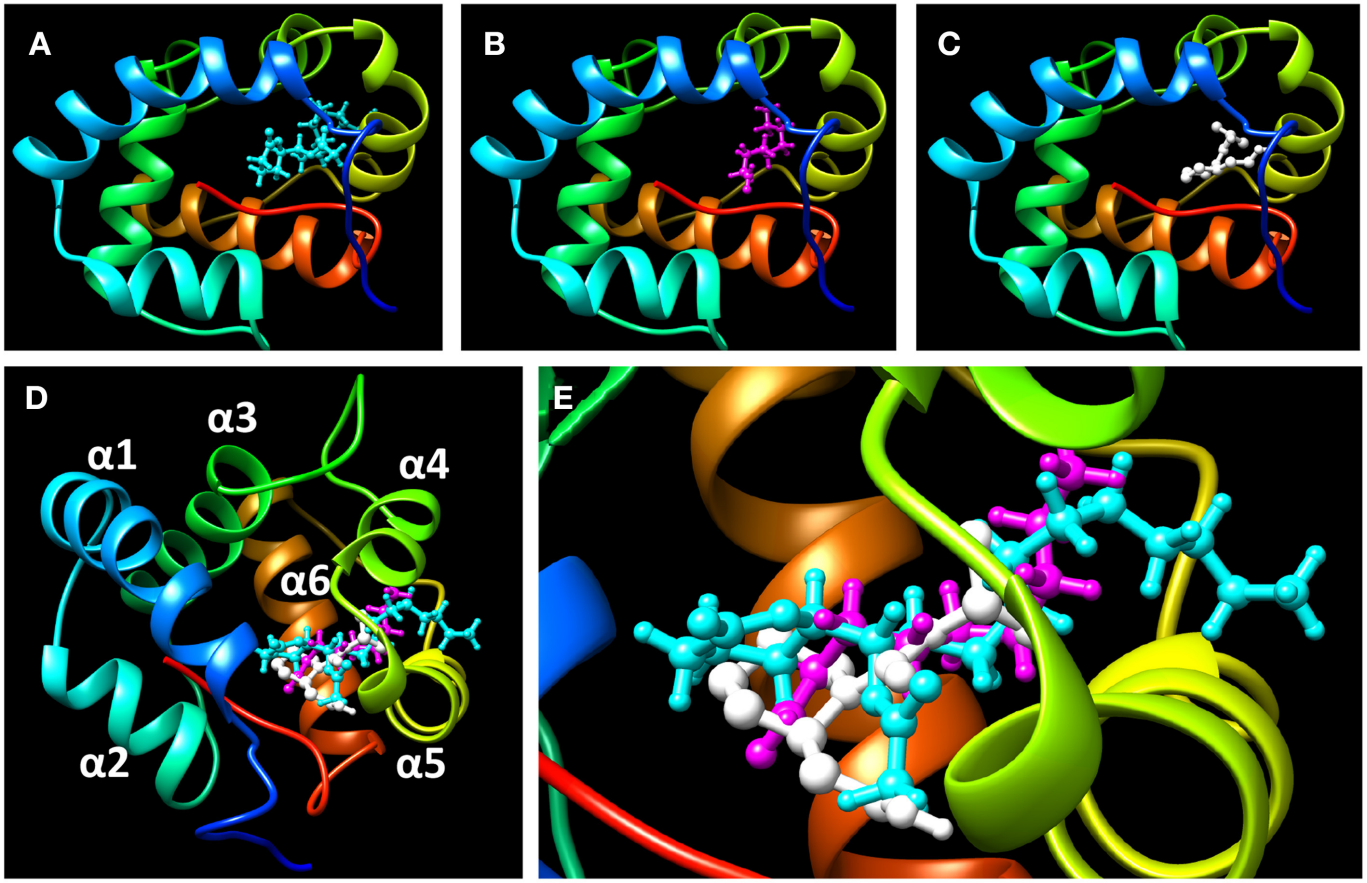
**Highlights of the ligands docked in the binding cavity of CquiOBP1**. **(A)** MOP, **(B)** nonanal, and **(C)** picaridin. Note that snapshots from **(A–C)** have the same OBP orientation. **(D)** the three docked ligands are overlapped: MOP (cyan), nonanal (magenta), and picaridin (white). Note that MOP polar head (lactone/acetate moiety) is housed in the central binding cavity and a large part of the hydrophobic chain is bound in the hydrophobic tunnel formed between helices 4 and 5. **(E)** Close up view of **(D)** Note that a segment of MOP about 5-carbon longer than the other ligands sticks inside the hydrophobic tunnel. CquiOBP1 is displayed in Chimera with rainbow-colored scheme from N-terminus in blue to C-terminus in red.

To assess the suitability of dock simulations in providing insights into the interactions of nonanal and picaridin with CquiOBP1, we first re-docked MOP into CquiOBP1 and compared this structure with the previously reported crystal structure (Figure 4A). The position of the polar moiety of MOP in the binding pocket matched that observed in the crystal structure. As previously described, MOP has its long lipid “tail” bound in a hydrophobic tunnel formed between helices 4 and 5 and only its lactone/acetate head is housed in the central cavity (Mao et al., 2010). There was a slight difference between the position of the hydrophobic moiety of MOP in the simulated and crystal structures, but this part of the molecule is flexible and different conformations could be accommodated in the hydrophobic tunnel. Docked nonanal was stabilized by a hydrogen bond between the oxygen atom of the carbonyl as a hydrogen bond acceptor and the (-NH-) group of Phe-123 in the backbone as hydrogen bond donor. The short hydrophobic tail of nonanal was only partially accommodated in the hydrophobic tunnel (Figure 4B). Interestingly, nonanal bound at the periphery of the central cavity even further away from the center than MOP (Figures 5A,B). Likewise, picaridin was accommodated at a similar location (Figure 5C), but having a different orientation. The polar moiety of picaridin was stabilized in the central binding cavity by a hydrogen bond between the hydroxyl group of picaridin (acceptor) and the side chain of Tyr-10 (donor) (Figure 4C). Despite the fact that all ligands are accommodated in the same binding pocket the orientations of the ligands in the binding pocket were different (Figures 5D,E). Additionally, MOP occupied the hydrophobic channel more so than the other ligands (Figure 5E). That MOP fits better in the binding cavity of CquiOBP1 is reflected in the Cscore of 8.51, as compared to 5.55 and 5.47 for picaridin and nonanal, respectively. These scores correlate well with the above-described dissociation constants.

## Conclusion

Using a fluorescence reporter and a panel of 34 physiologically relevant compounds, we measured binding affinities of three major OBPs from the southern house mosquito, namely, CquiOBP1, CquiOBP2, and CquiOBP5. Based on dissociation constants, we hypothesized that CquiOBP2 is a carrier for the oviposition attractant skatole, and CquiOBP1 and CquiOBP5 might transport the oviposition pheromone MOP, a human-derived attractant nonanal, and the insect repellent picardin. Examination of binding of these three ligands to CquiOBP1 at various pH values suggests that CquiOBP1 might discriminate MOP from nonanal/picaridin on the basis of the midpoint transition of a pH-dependence conformational change. Additionally, docking studies suggest MOP is better accommodated in the binding cavity than the other two ligands. Taken together, these findings suggest that OBPs may be involved in the selectivity of the mosquito olfactory system, but this may not be manifested clearly in binding affinities.

### Conflict of interest statement

The authors declare that the research was conducted in the absence of any commercial or financial relationships that could be construed as a potential conflict of interest.
